# Diagnostic Challenges in Inflammatory Choroidal Neovascularization

**DOI:** 10.3390/medicina60030465

**Published:** 2024-03-12

**Authors:** Izabella Karska-Basta, Weronika Pociej-Marciak, Katarzyna Żuber-Łaskawiec, Anna Markiewicz, Michał Chrząszcz, Bożena Romanowska-Dixon, Agnieszka Kubicka-Trząska

**Affiliations:** 1 Chair of Ophthalmology, Faculty of Medicine, Medical College, Jagiellonian University, Kopernika Str. 38, 31-501 Krakow, Poland; izabella.karska-basta@uj.edu.pl (I.K.-B.); anna.markiewicz@uj.edu.pl (A.M.);; 2 Clinic of Ophthalmology and Ocular Oncology, University Hospital, Kopernika Str. 38, 31-501 Krakow, Poland; m.a.chrzaszcz@gmail.com

**Keywords:** uveitis, inflammatory choroidal neovascularization, multimodal imaging, fluorescein angiography, indocyanine green angiography, optical coherence tomography, optical coherence tomography angiography, near-infrared autofluorescence

## Abstract

Inflammation plays a key role in the induction of choroidal neovascularization (CNV). Inflammatory choroidal neovascularization (iCNV) is a severe but uncommon complication of both infectious and non-infectious uveitides. It is hypothesized that its pathogenesis is similar to that of wet age-related macular degeneration (AMD), and involves hypoxia as well as the release of vascular endothelial growth factor, stromal cell-derived factor 1-alpha, and other mediators. Inflammatory CNV develops when inflammation or infection directly involves the retinal pigment epithelium (RPE)–Bruch’s membrane complex. Inflammation itself can compromise perfusion, generating a gradient of retinal–choroidal hypoxia that additionally promotes the formation of choroidal neovascularization in the course of uveitis. The development of choroidal neovascularization may be a complication, especially in conditions such as punctate inner choroidopathy, multifocal choroiditis, serpiginous choroiditis, and presumed ocular histoplasmosis syndrome. Although the majority of iCNV cases are well defined and appear as the “classic” type (type 2 lesion) on fluorescein angiography, the diagnosis of iCNV is challenging due to difficulties in differentiating between inflammatory choroiditis lesions and choroidal neovascularization. Modern multimodal imaging, particularly the recently introduced technology of optical coherence tomography (OCT) and OCT angiography (noninvasive and rapid imaging modalities), can reveal additional features that aid the diagnosis of iCNV. However, more studies are needed to establish their role in the diagnosis and evaluation of iCNV activity.

## 1. Introduction

Uveitis-related choroidal neovascularization (CNV), known as inflammatory CNV (iCNV), is an uncommon complication of uveitis. At the same time, it is one of the most severe causes of visual impairment in patients with uveitis [1,2,3]. Inflammatory CNV is the third cause of CNV following wet age-related macular degeneration (AMD) and pathological myopia [2,3]. Inflammatory CNV develops more frequently in patients with posterior uveitis (2.7%) and panuveitis (0.8%) as compared with those with anterior and intermediate uveitis (0.1%) [4]. CNV is a clinically important complication of both infectious and non-infectious uveitis and is found to be more frequent in some specific clinical entities. Its incidence in non-infectious posterior uveitis has been reported to reach 2% [5]. A higher incidence of CNV was reported in multifocal choroiditis, punctate inner choroidopathy, serpiginous choroidopathy, birdshot retinochoroidopathy, and Vogt–Koyanagi–Harada disease [6,7,8,9,10,11,12,13,14,15,16,17,18]. On the other hand, data on the incidence of iCNV secondary to infectious uveitis are scarce and are derived mainly from case series and case reports. The prevalence of iCNV, depending on the etiology of uveitis, is presented in Table 1. 

According to Baxter et al. [4], the presence of epiretinal neovascularization is associated with an over three-fold higher risk of iCNV. Moreover, the risk of CNV was significantly greater in eyes with active inflammation vs. those with inactive inflammation. The presence of anterior chamber cells grade 2+, as defined by the Standardization of Uveitis Nomenclature Working Group, was shown to be associated with iCNV. However, increased vitreous cells and vitreous haze were not significantly associated with an altered risk of incident CNV in patients with uveitis [4]. A previous diagnosis of CNV in the contralateral eye was associated with a several-fold higher risk of CNV in the second eye. However, the eyes of patients with bilateral uveitis were less likely to develop CNV than the affected eyes of patients with unilateral uveitis [4]. 

The underlying pathophysiology of iCNV is likely similar to the pathophysiology of CNV in other conditions associated with CNV, such as age-related macular degeneration (AMD) or pathologic myopia. Inflammatory CNV may thus be considered not as a distinct form of CNV, but rather as associated with a set of circumstances that permit its development [1,2,3].

It was demonstrated that vascular endothelial growth factor (VEGF) plays a key role in CNV development [2,3]. Moreover, CNV has an extravascular component consisting of fibroblasts and leukocytes that express C-X-C motif chemokine receptor 4 (CXCR4). Retinal pigment epithelial (RPE) cells showed an increased production of tumor necrosis factor as well as interleukins IL-1, IL-2, IL-6, and IL-10, accounting for the inflammatory component of CNV [47,48]. Also, other mediators are involved in CNV development, such as nitric oxide, angiostatin, endostatin, C-C chemokine receptor type 3 (CCR3), and pigment epithelium-derived growth factor (PEDF), contrasting the neovascularization [49]. 

Recently, D’Ambrosio et al. [8], based on the immunohistochemical staining grading of three structures of CNV, namely, the RPE, vascular network, and fibroblasts for SDF1, CXCR4, and VEGF receptor 2, revealed differences in the CXCR4 staining of the vascular meshwork of iCNV as compared with AMD-related CNV, suggesting that capillaries have a different role in membrane development [8]. However, because of a low P value for the CXCR4 staining of the vascular meshwork of uveitis-related CNV versus AMD-related CNV, the authors concluded that further studies on this distinctive aspect are necessary [8].

There are two pathophysiological mechanisms by which uveitis can promote the development of iCNV [2,47]. The first one is associated with inflammation-mediated damage of the RPE-Bruch’s membrane complex, which disrupts the outer blood-retinal barrier and permits neovascular upgrowth from the choroid. The damage can be induced by an angiogenic stimulus mediated by local inflammation, or it can result from a combination of both [2]. The imbalance between the inhibitory and stimulatory actions of the soluble mediators produced by the RPE is supposed to be the trigger of neoangiogenesis. Activated inflammatory cells secrete enzymes that damage cells and cause degradation in the Bruch’s membrane. Proangiogenic cytokines released by these inflammatory cells may promote CNV growth through breaks in the membrane and into the sub-RPE space, potentially leading to edema, exudation, hemorrhages, and fibrosis, resulting in profound central vision loss [1,2]. Thus, a higher risk of damage to the RPE–Bruch’s membrane complex related to adjacent chorioretinal inflammation may explain the higher incidence of iCNV in patients with panuveitis and posterior uveitis [50]. It is noteworthy that the RPE is often intact in individuals with iCNV, and a majority of iCNVs are type 2 lesions (“classic” type CNVs) with abnormal growth of the vasculature into the outer retinal space [2,8]. The proposed mechanism of iCNV development is thus the focal breach of the RPE due to infection or inflammation leading to the growth and entry of new vessels into the outer retinal space [50]. The second theory explains that retinal and choroidal inflammation directly compromises perfusion, generating a gradient of retinal–choroidal hypoxia that promotes the formation of choroidal neovascularization [2]. It should also be noted that, in some cases, “idiopathic” choroidal neovascularization may herald the subsequent development of posterior uveitis [51]. 

Inflammatory CNV is typically diagnosed when a patient with uveitis reports a sudden deterioration of vision and/or metamorphopsias [2,3,4,52]. In advanced cases of iCNV, a central scotoma may be present. Some active extrafoveal lesions can be asymptomatic and may be missed initially due to the presence of associated features such as inflammatory lesions, scars, and pigmentation, as well as intra- or subretinal fluid accumulation [1,2,3,8]. In such cases, iCNV may be detected in imaging studies [1,6,52,53]. 

Inflammatory CNV lesions typically grow close to the edge of a postinflammatory atrophic chorioretinal scar, although iCNV can rarely be synchronous with an active disease [52,53]. Inflammatory CNVs can be subfoveal, extrafoveal, or juxtafoveal, and are highly focal [8]. These can be associated with intra- or subretinal hemorrhages and exudations. Inactive iCNV can result in a subretinal yellow-white scar, which may be associated with fibrosis and pigmentation. The presence of intra- or subretinal fluid with iCNV, as well as serous retinal detachment, may also represent signs of inflammation, leading to a misdiagnosis during a basic ophthalmological examination [1,2,3,4,8].

Thus, in patients with posterior uveitis, the identification of iCNV is challenging due to related abnormalities, including choroiditis, chorioretinal scarring, and inflammatory lesions [52,53]. Difficulties in a differential diagnosis of iCNV and active inflammatory lesions arise from the characteristic presence of intraretinal or subretinal fluid as well as serous retinal detachment in both [1,8,52]. An accurate characterization of inflammatory lesions is important for the diagnosis of an underlying pathology and the implementation of adequate treatment. 

The aim of this paper was to discuss current multimodal imaging tools for the diagnosis of iCNV, with an emphasis on technological advances and future perspectives.

## 2. Imaging Tools for the Detection of iCNV

### 2.1. Fluorescein Angiography

**Figure 1:**
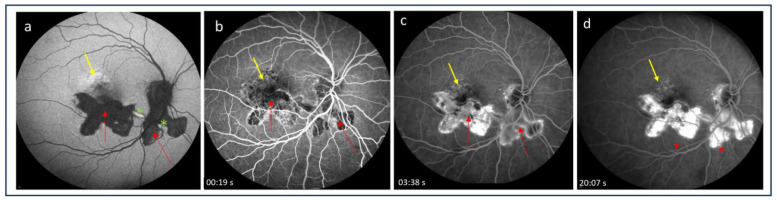
(**a**) Fundus autofluorescence image, showing hypoautofluorescence of inactive postinflammatory lesions (red arrows) and hyperautofluorescence of active inflammatory lesions (green asterix); irregular autofluorescence is present above the postinflammatory scar suspected for inflammatory choroidal neovascularization (yellow arrow); (**b**,**c**) fluorescein angiography images, showing early isofluorescence with late staining (**d**) of the lesions as a result of a retinal pigmented epithelium window defect; no evident features of inflammatory choroidal neovascularization are observed.

**Figure 2:**
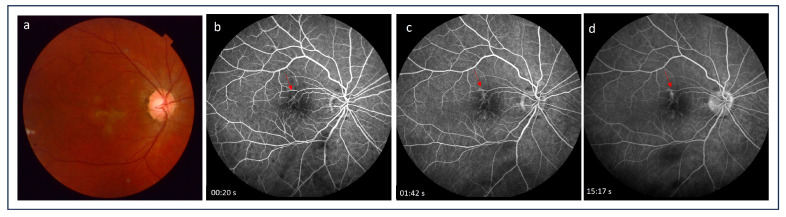
(**a**) Color picture of the fundus showing inactive inflammatory foci in the macula region with changes at the retinal pigmented epithelium level; (**b**,**c**) fluorescein angiography images demonstrate early isofluorescence with late staining (**d**) of the lesions as a result of a retinal pigmented epithelium window defect without features of inflammatory choroidal neovascularization (red arrows).

Fluorescein angiography (FA) has been widely employed in the diagnosis of CNV secondary to various ocular pathologies. Since iCNV is often a classic type of neovascular membrane (type 2), it can be visualized by FA. CNV lesions are present on FA as early iso- or hyperfluorescence with late leakage [54]. Similarly, active inflammatory lesions show features of early isofluorescence (although mostly hypofluorescence) and late leakage, while inactive atrophic lesions are characterized by early hypo- or isofluorescence with late staining (suggesting an RPE window defect) without leakage [4,55,56] (Figure 1 and Figure 2). These highly similar FA features of iCNV and inflammatory lesions pose a diagnostic challenge. In conditions such as multifocal choroiditis, serpiginous choroiditis, or Vogt–Koyanagi–Harada disease, which present with scarring and pigmentation due to extensive retinal involvement, the detection of hyperfluorescence associated with CNV may be particularly difficult [52,53,57]. Thus, FA alone may be insufficient to identify iCNV lesions and initiate appropriate therapy. Therefore, a multimodal approach with additional tests is recommended. 

### 2.2. Indocyanine Green Angiography

**Figure 3:**
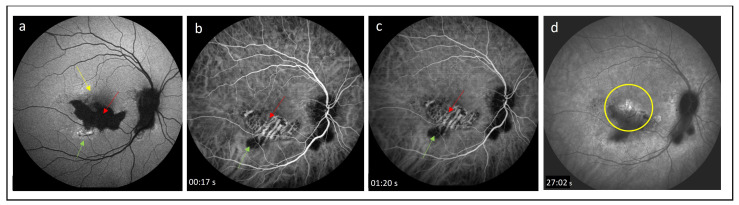
(**a**) Fundus autofluorescence image shows hypoautoflurescence of inactive postinflammatory lesion in macula (red arrow) with hyperautofluorescent area representing the active inflammatory focus at its lower margin (green arrow), at the upper border of the scar the area of irregular autofluorescence is present (yellow arrow); (**b**,**c**) early and middle frames of indocyanine green (ICG) angiograms reveal a visibility of the choroidal vessels in the area of the inactive postinflammatory focus and small hypocyanescent area at its lower border corresponding to active choroidal inflammation; (**d**) late ICG angiogram demonstrates homogeneous hypocyanescence of the postinflammatory lesion with evidence of late hypercyanescence at its upper margin (yellow circle). The patient was diagnosed with inflammatory choridal neovascularization.

**Figure 4:**
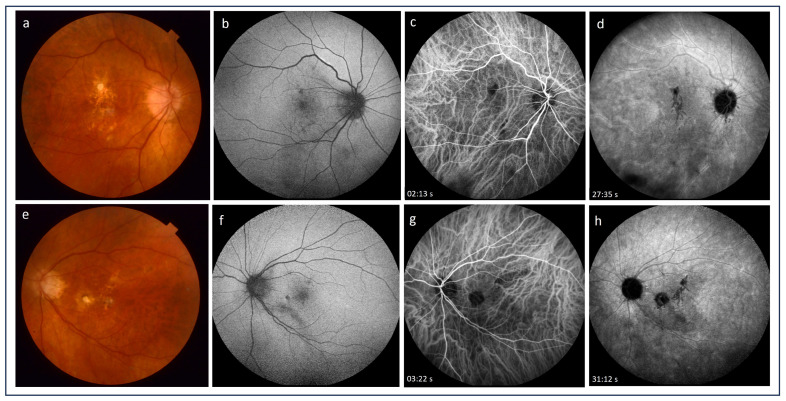
(**a**,**e**) Color pictures of the fundus presenting well-defined and partially pigmented chorioretinal scars at the sites of previous chorioretinal inflammation in the macula of both eyes; (**b**,**f**) fundus autofluorescence demonstrate hypoautofluorescent foci that correlate with photoreceptor and retinal pigment epithelium damage; (**c**,**d**,**g**,**h**) early and late frames of indocyanine green angiograms revealed the presence of homogenous hypocyanescent foci related to retinal pigment epithelium damage due to postinflammatory scarring; no features of inflammatory choroidal neovascularization are observed.

One of the imaging techniques for the visualization of the choroid is indocyanine green angiography (ICGA), which allows a better visualization of the choroid compared with FA [58]. ICGA plays an important role in assessing pathologies involving the choroidal vasculature and choriocapillaris in chorioretinal inflammatory diseases. It is helpful in differentiating between iCNV and inflammatory lesions [59,60]. ICGA allows us to tell the difference between a recurrent inflammatory focus and iCNV: the former appears as an early hypofluorescent lesion, whereas the latter has been a hyperfluorescent lesion since early angiographic frames [58,59]. ICGA is also mandatory in the case of CNV associated with choriocapillaritis, such as multifocal choroiditis (MFC), where it shows the extent of occult choriocapillaris nonperfusion and hence the risk for CNV development [60,61]. CNV secondary to MFC is more frequent in inflamed areas; however, this may originate from an old chorioretinal scar as well. Low-grade chronic inflammation can be at the core of this process, and ICGA frequently shows areas of non-perfusion indicating ischemia, which may be the trigger of angiogenesis. In such cases, ICGA is essential in the evaluation of the choroidal status. Importantly, ICGA was shown to outperform FA in detecting occult CNV lesions [54]. While iCNV is typically a classic lesion that can be easily visualized by FA, ICGA has been recently reported to be more accurate in assessing the size of neovascular lesions, especially in patients with idiopathic CNV, which shares several clinical features with iCNV [62]. Thus, ICG helps identify both iCNV and inflammatory choroidal alterations in patients with uveitis, allowing clinical differentiation between these lesions and a more comprehensive evaluation of the disease [60] (Figure 3 and Figure 4). 

### 2.3. Optical Coherence Tomography 

Optical coherence tomography (OCT) is a noninvasive and highly repeatable imaging technique that has revolutionized the management of retinal and choroidal diseases. By providing the quasi-histological sections of the ocular structure, it allows clinicians to identify ocular pathologies and assess response to treatment [63]. Moreover, the enhanced depth imaging modality of OCT can be used to evaluate choroidal thickness and structural modifications, which is particularly valuable in the treatment of uveitis [63]. 

**Figure 5:**
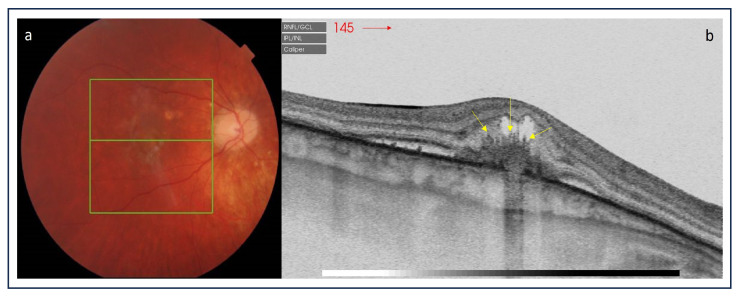
(**a**) Color picture of the right eye diagnosed for punctate inner choroidopathy; (**b**) optical coherence tomography scan demonstrates hyperreflective material above the retinal pigment epithelium with the presence of subretinal and intraretinal fluid corresponding to an active inflammatory choroidal neovascularization with multiple vertical finger-like projections extending anteriorly into the outer retina—the “pitchfork sign” (yellow arrows).

Inflammatory CNV usually develops between the RPE and neurosensory retina, demonstrating similar features on OCT imaging as classic (type 2) CNV [58]. In both cases, the lesions appear as hyperreflective structures located in front of a disrupted RPE, with solid tissue in the subretinal space [1,64,65,66]. However, there is a single OCT feature that can help distinguish between iCNV and other classic CNVs. This is the so-called “pitchfork sign”, characterized by finger-like hyperreflective lesions extending from the CNV into the outer retinal layers, and it allows us to differentiate iCNV from other causes of CNV [1,53,67,68] (Figure 5). The inflammatory conditions associated with this sign include idiopathic multifocal choroiditis/punctate inner choroidopathy (MFC/PIC), intraocular tuberculosis, and acute syphilitic posterior placoid chorioretinitis [1,53,67,68]. Rajabian et al. and Berensztejn et al. reported “pitchfork signs” in patients with choroidal osteoma. The authors proposed that inflammation is the most important stimulus for the development of CNV in these cases [69,70]. However, recently, Falavrajani et al. [71] have described the “pitchfork sign” in five eyes with type 2 CNV and without any sign of ocular inflammation. They speculated that traction of the type 2 CNV complex on the outer retinal layers and consequent dragging of the layers or Müller cell activation could explain the presence of the “pitchfork sign” [71]. 

**Figure 6:**
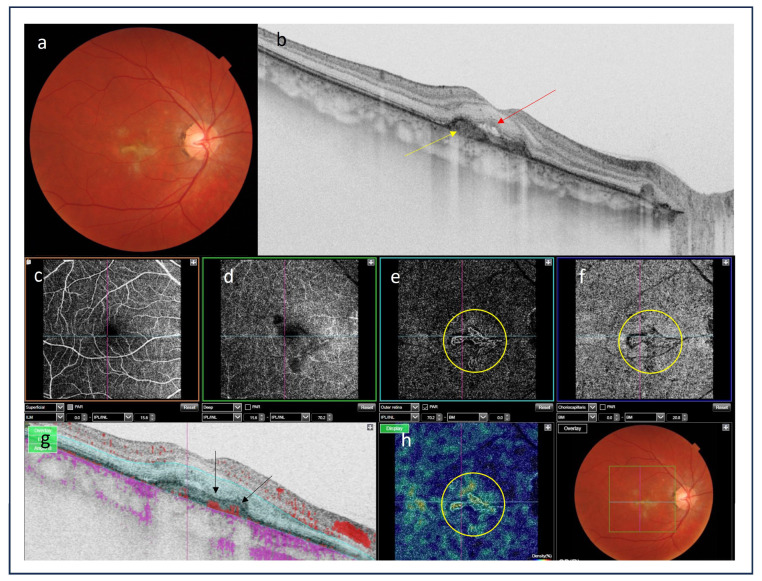
(**a**) Color picture of the fundus shows macular subretinal fibrosis; (**b**) optical coherence tomography scan revealed hyperreflective material under the retinal pigment epithelium (yellow arrow), subretinal fluid is present (red arrow); (**c**–**f**) optical coherence tomography angiography (OCTA): the scan of the superficial capillary plexus is undisrupted (**c**); the scan of the deep capillary plexus shows a capillary impairment (**d**); the scans of outer retinal layer and choriocapillaris demonstrate pathological vessels presenting inflammatory choroidal neovascularization (iCNV) (yellow circles); (**g**) OCTA scan B presents the blood-flow in outer retinal layer (black arrows); (**h**) OCTA vessel density map is complementary and shows the presence of pathological vessels corresponding to iCNV (yellow circle).

It was reported that the OCT features of CNV activity such as retinal thickening, subretinal and intraretinal fluid, intraretinal hyperreflective flecks, and undefined boundaries of subretinal material predicted the presence of FA leakage [1,66] (Figure 6b,g). Thus, it was concluded that OCT can be used for monitoring disease progression and response to treatment [68,72].

**Figure 7:**
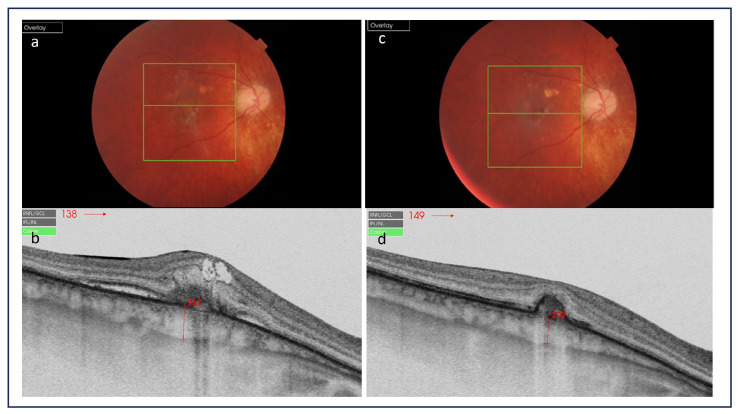
(**a**,**c**) Color pictures of the right eye diagnosed for punctate inner choroidopathy; (**b**,**d**) optical coherence tomography scans demonstrate choroidal thickness (CT) changes under inflammatory choroidal neovascularization before and after the treatment—the “sponge sign”. Before treatment, CT corresponded to 257 µm and then decreased to 202 µm after intravitreal bevacizumab injection.

Moreover, central retinal thickness evaluated by OCT is often used as an objective measure of iCNV activity [73,74,75]. Recently, Giuffrè et al. [76] demonstrated increased choroidal thickness under iCNV that decreases after therapy: the so-called “sponge sign” (Figure 7). The authors investigate choroidal thickness changes related to the clinical activity of inflammatory choroidal neovascularization in punctate inner choroidopathy/multifocal choroiditis as compared to myopic choroidal neovascularization. They found that choroidal thickness beneath inflammatory choroidal neovascularization significantly increased at baseline and decreased after therapy, reaching preclinical values. Conversely, no significant choroidal thickness changes were disclosed in myopic choroidal neovascularization eyes, under any location. Thus, OCT-based choroidal thickness evaluation may represent an additional useful tool to monitor inflammatory choroidal neovascularization activity. Moreover, choroidal thickness under CNV could be used to discriminate the origin of the choroidal neovascular membrane in doubtful cases (either inflammatory or myopic) and to guide therapeutic management [76]. 

OCT images can also help differentiate between iCNV lesions and non-neovascular alterations at the RPE level that are characteristic of several types of uveitis. For example, acute inflammatory foci in multifocal choroiditis show a deeper penetration of the OCT signal, a feature that is usually not seen in iCNV [56,65]. However, when distinguishing CNV lesions from iCNV, the use of OCT alone may be limited as these lesions display similar features of outer retinal or RPE hyperreflectivity, intraretinal edema, sub-RPE fluid, and exudation in conditions with the involvement of the RPE or choriocapillaris (e.g., multifocal choroiditis and punctate inner choroidopathy) [1,74,75,77,78]. In such cases, the characteristics of the lesions can be determined by a combination of FA, ICGA, and OCT angiography (OCTA). 

### 2.4. Optical Coherence Tomography Angiography 

**Figure 8:**
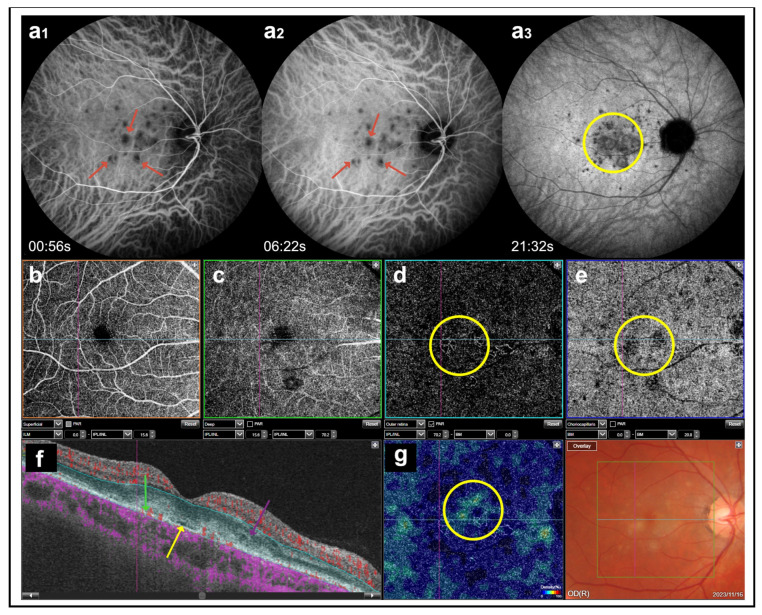
(**a_1_**,**a_2_**) Early and middle stages of indocyanine green angiography present hypocyanescent spots indicating active inflammatory lesions (red arrows); (**a_3_**) late stage shows hypercyanescent plaque (green circle); (**b**–**e**) optical coherence tomography angiography (OCTA): the scan of the superficial apillary plexus is undisrupted (**b**); the scan of the deep capillary plexus shows a small area of capillary impairment below the macula (**c**); the scans of the outer retinal layer and choriocapillaris demonstrated pathological vessels presenting inflammatory choroidal neovascularization (green circles) (**d**,**e**); (**f**) OCTA B scan shows the presence of a trace of subretinal fluid (yellow arrow), blood flow in the outer retinal layers (green arrow), and intraretinal fluid (pink arrow), indicating the presence of active inflammatory choroidal neovascularization; (**g**) OCTA vessel density map presents a net of pathological vessels (yellow circle).

**Figure 9:**
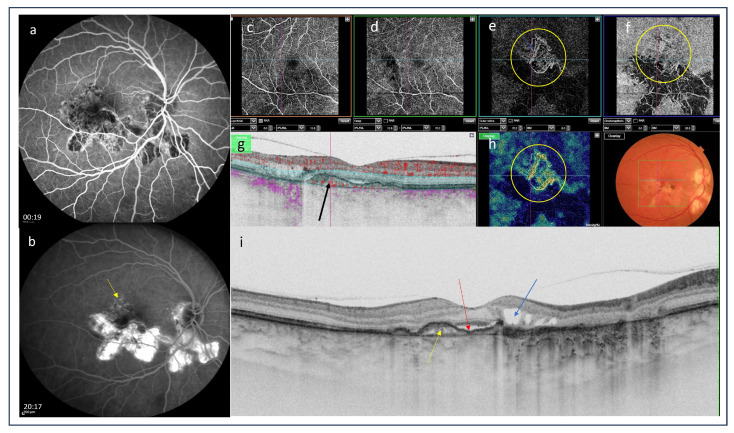
(**a**) Early frame of fluorescein angiography demonstrates the presence of isofluorescence of the inactive inflammatory lesion related to serpiginous choroidopathy; (**b**) Late frame of fluorescein angiography presents hyperfluorescent area of inactive postinflammatory lesion as a result of a “window defect” with irregular staining above it (yellow arrow); (**c**–**f**) Optical coherence tomography angiography demonstrated normal superficial and deep capillary plexi (**c**,**d**); the scans of outer retina and choriocapillary layers show the net of pathological vessels (**e**,**f**); (**g**) OCTA B scan shows the presence of subretinal mass with blood flow (black arrows); (**h**) OCTA vessel density map presents a net of pathological vessels (yellow circle); (**i**) Optical coherence tomography presents the hyperreflective subretinal lesion (yellow arrow) with subretinal fluid (red arow) and intraretinal fluid (blue arrow) showing the evidence of active inflammatory choroidal neovasularization.

The usefulness of OCTA as a noninvasive technique for the detection of iCNV has been reported by several investigators. Cheng et al. [79] assessed the ability of OCTA to detect iCNV and differentiate it from inflammatory lesions as compared with conventional FA in 26 patients with multifocal choroiditis. The authors concluded that OCTA outperformed FA in differentiating CNV from inflammatory lesions, as the latter do not show any blood flow signals. It also permitted the visualization of the detailed vascular structure of CNV. Therefore, it could be used as an alternative option for CNV identification and to guide therapeutic decision making [77]. Similarly, in a recent retrospective study of 14 patients, Zahid et al. [80] used OCTA to evaluate neovascular flow signals in macular chorioretinal lesions occurring in idiopathic multifocal choroiditis. They concluded that OCTA may be a useful tool for understanding the pathophysiology of the disease and monitoring its course. The utility of OCTA for the noninvasive diagnosis of iCNV and its subsequent follow-up was also confirmed by Yee et al. [81]. Finally, in a recent study, Aggarwal et al. [82] investigated the OCTA features of tuberculosis-associated choroiditis in comparison with conventional imaging modalities, including FA, ICGA, and OCTA. This was the first study to demonstrate that OCTA can identify type 1 neovascular networks. The research led to the conclusion that OCTA is indispensable to exclude neovascular networks when FA, ICGA, and OCT results are inconclusive [81] (Figure 6c–h, Figure 8 and Figure 9).

In patients with posterior uveitis, the identification of iCNV is challenging due to related abnormalities, including associated pathologies such as choroiditis, chorioretinal lesions, and choroidal scarring [1,82,83]. In such cases, OCTA allows noninvasive diagnostic imaging of iCNV and differentiation from inflammatory pathologies [1,82,83].

While the above studies prove the role of OCTA in the diagnosis and follow-up of patients with iCNV, larger prospective studies are needed to determine its advantages over conventional imaging. 

### 2.5. Near-Infrared Autofluorescence Imaging

Fundus autofluorescence (FAF) is a valuable imaging tool for multiple anatomical and physiological alterations in the ocular tissue [84]. Essentially, FAF is a map of lipofuscin distribution, which is the autofluorescent pigment of the eye naturally found in the RPE–photoreceptor complex [85]. It was reported that iCNV lesions may show a different pattern on FAF imaging than active inflammatory foci [1,84,85]. In FAF, normal autofluorescence may occur in active iCNV with preserved neurosensory retina [1,83,84,85,86]. Prolonged active CNV tends to present hyperautofluorescence, while hypoautofluorescent areas correlate with photoreceptor and RPE loss [1,83,86] (Figure 1a and Figure 7b). Active inflammatory foci may show an increased autofluorescence signal [84,85] (Figure 3a). Therefore, the technique may be used for differentiating between inflammatory and CNV lesions.

## 3. Conclusions

The detection of inflammatory CNV remains a challenge due to the presence of choroiditis lesions, scarring, and pigmentation that make it difficult to visualize CNV lesions. Available case reports and case series point to the benefits of using OCTA in combination with conventional imaging modalities such as FA, OCT, and ICGA. This multimodal approach may improve the detection and follow-up of iCNV lesions. OCTA is also an important addition to FA and ICGA in terms of providing important information on retinochoroidal abnormalities associated with uveitis, such as the severity of inflammation or the presence of any vascular changes and focal lesions. It may offer advantages over traditional modalities in the detection of neovascular flow in uveitis, but it should be used as an additional tool rather than a replacement for the existing ones. Also, FAF requires further research to reveal whether this imaging modality can be used to differentiate between iCNV and inflammatory lesions and between AMD-related CNV and CNV related to inflammatory disease. 

## Figures and Tables

**Table 1:** Prevalence of inflammatory choroidal neovascularization (iCNV) depending on the etiology of uveitis.

Type of Uveitis	Prevalence of iCNV [References]
**Non-infectious**	
Multifocal choroiditis	32–50% [4,5,6,7]
Punctate inner choroidopathy	17–40% [1,4,8,9]
Serpiginous choroidopathy	10–25% [4,10,11]
Vogt–Koyanagi–Harada disease	9–15% [4,7,9,12]
Birdshot chorioretinitis	5% [4,13]
Intermediate uveitis	single cases reported [14,15]
Behçet disease	very rare [4,16]
Acute posterior multifocal placoid pigment epitheliopathy	single cases reported [4,16,17,18]
Sympathetic ophthalmia	single cases reported [16,19]
Sarcoidosis	single cases reported [4,9,16]
Multiple evanescent white dot syndrome	single cases reported [4,20,21]
**Infectious**	
Histoplasmosis	5–17.4% [4,9,16,22,23,24,25]
Candidiasis	rare; prevalence unknown [26,27,28]
Toxoplasmosis	0.3–19% [29,30,31,32]
Toxocariasis	single cases reported [33,34,35]
Tuberculosis	single cases reported [36,37,38,39]
Rubella retinopathy	single cases reported [40,41,42,43]
West Nile virus	single cases reported [44,45,46]

## Data Availability

Data supporting the findings of this study are available upon request from the corresponding author.
